# Para-Aortic Lymphadenectomy in Ovarian, Endometrial, Gastric, and Bladder Cancers: A Systematic Review of Randomized Controlled Trials

**DOI:** 10.3390/cancers16193394

**Published:** 2024-10-04

**Authors:** Souhail Alouini, Younes Bakri

**Affiliations:** 1 Department of Gynecological Surgery and Obstetrics, CHU Orleans, 14 Avenue de L’hôpital, 45100 Orleans, France; 2 Département Médecine, Université d’Orléans, Château de la Source Avenue du Parc Floral, 45067 Orleans, France; 3 Department of Surgery Oncology, Ayoub Medical Center, Amman 11931, Jordan; bakri.balloon@bakrimedical.com

**Keywords:** para-aortic lymphadenectomy, overall survival, ovarian cancer, bladder cancer, endometrial cancer, gastric cancer

## Abstract

**Simple Summary:**

Para-aortic lymphadenectomy can be used for both staging and therapeutic purposes in ovarian, endometrial, gastric and bladder cancers. However, Para-aortic lymphadenectomy is associated with high rates of morbidity and mortality and with no evidence of benefits in terms of overall survival and disease-free survival. A systematic review of the literature was performed to look into the published randomized controlled studies (RCTs) that have reported the effectiveness of lymphadenectomy. The total number of patients was 4231. The studies reported that para-aortic lymphadenectomy did not improve overall survival and disease-free survival in advanced ovarian cancers, early endometrial cancers, advanced gastric, and bladder cancers. All of the studies had a low risk of bias. Conclusions: Para-aortic lymphadenectomy is not advised in advanced ovarian cancers, early endometrial cancers with low risks, advanced gastric cancers, and bladder cancers. Clinicians should inform patients regarding the benefits of para-aortic lymphadenectomy in terms of survival and the potential risks associated with it.

**Abstract:**

Background: Para-aortic lymphadenectomy can be used for both diagnostic and therapeutic purposes as it aids in staging, provides prognostic data, and influences the patient’s options for adjuvant therapy. However, there is still contention over its potential in treating cancer. A systematic review of the literature was performed to look into the published randomized controlled studies (RCTs) that have reported the effectiveness of lymphadenectomy. Methods: Five different electronic databases, including PubMed, Cochrane Library, Clinical trials.gov, ICTRP, and Embase, were used to conduct a comprehensive search. Original RCTs reporting on the impact of lymphadenectomy on the overall survival in various cancers were included. Information related to the study population, intervention, type of cancer, primary endpoints, and key findings of the study were extracted. Quality assessment of the selected studies was conducted using the Revised Cochrane Risk of Bias Tool Rob 2 for randomized trials. Results: A total of 1693 citations, with 1511 from PubMed, 80 from the Cochrane Library, 67 from Embase, 18 from ICTRP, and 17 from Clinicaltrials.gov were retrieved. Preliminary screening was performed, and after applying selection criteria, nine articles were included in the final qualitative analysis. The total number of patients was 4231, and the sample size ranged from 70 to 1408. Among these nine studies, four studies were on genital cancers (two ovarian cancers, one endometrial cancer, and one cervical cancer); four on digestive cancers (advanced gastric cancers); and one on urinary cancer (advanced bladder cancer). These studies reported that para-aortic lymphadenectomy did not improve overall survival and disease-free survival in advanced ovarian cancers, early endometrial cancers, advanced gastric, and bladder cancers. All of the studies had a low risk of bias. Conclusions: Para-aortic lymphadenectomy is not advised in advanced ovarian cancers, early endometrial cancers with low risks, advanced gastric cancers, and bladder cancers. SNB could be an alternative to lymphadenectomy for ovarian cancer in the future. Clinicians should inform patients regarding the benefits of para-aortic lymphadenectomy in terms of survival and the potential risks associated with it.

## 1. Introduction

The cornerstone of advanced cancer treatment is primary surgery aiming to completely remove all macroscopically apparent tumors, followed by chemotherapy. The treatment is deemed effective if there are no macroscopically visible tumor remains after excision [1,2]. However, lymphatic spread is a common feature and a critical prognostic factor in both early and late cancers. Lymph node metastasis between the aortic hiatus and the aortic bifurcation is known as a para-aortic lymph node metastasis [1]. Studies of unselected case series encompassing patients with disease in all International Federation of Gynecology and Obstetrics (FIGO) stages have shown a 44–53% rate of lymph node metastases that have been identified by systematic lymphadenectomy [3,4,5,6]. In 50–80% of the cases, patients with advanced ovarian cancer may have involvement of the retroperitoneal lymph nodes [3,7,8]. About 10% of women with clinical Stage I endometrial cancer, which is limited to the corpus, have pelvic lymph node metastases [2]. About 20–25% of patients who have radical cystectomy were found to have pelvic and/or para-aortic lymph node (LN) metastases, which is a risk factor for a poor oncologic outcomes [9,10]. Also, the risk of para-aortic lymph node (PAN) metastases is 17–40% in those with advanced gastric cancer [1].

Pelvic and para-aortic lymphadenectomy surgery is usually carried out by gynecologic oncologists [11]. It can be used for both diagnostic and therapeutic purposes. It aids in staging, provides prognostic data, and influences the patient’s options for adjuvant therapy. Lymphadenectomy can be necessary for endometrial cancer staging and ovarian cancer debulking. Lymphadenectomy can provide prognostic information even if it is not a component of cervical cancer staging [1,12]. The main objective of the treatment of advanced ovarian cancer is the macroscopically complete resection of all visible tumors, followed in most cases by chemotherapy that includes carboplatin and paclitaxel [4]. The prognosis of ovarian cancer depends on the size of the largest residual tumor present after surgery and lymph node involvement [3].

Some retrospective studies have reported a potential survival benefit of systematic pelvic and para-aortic lymphadenectomy in patients with macroscopically completely resected advanced ovarian cancer [13]. However, another prospective randomized study did not show an overall survival advantage [13,14]. The Lymphadenectomy in Ovarian Neoplasms (LION) trial [3] was a prospectively randomized trial.

Radical cystectomy with lymph node dissection is the mainstay of management for those with muscle-invasive bladder cancer [9]. According to data from the Gastric Cancer Registry and other retrospective research, radical gastrectomy with an expanded (D2) excision of local lymph nodes is the standard of care for the treatment of curable stomach cancer [1,4,15]. However, many surgeons continue to debate whether the super-extended lymph node dissection, D2 plus PAND, or the so-called D2+ resection is beneficial.

The therapeutic rationale for a regular para-aortic lymphadenectomy in the surgical management of gynecological malignancies is strongly debated. However, there are no studies that unequivocally demonstrate that lymphadenectomy is effective for ovarian, endometrial, gastric, and bladder cancers. Although management for local endometrial cancer and advanced ovarian cancer is often provided at facilities around the world, there is comparatively little evidence of its effectiveness. Patients who undergo systematic pelvic and para-aortic lymphadenectomies need more extensive surgery and are more likely to experience complications during and after surgery [3,9,10].

This systematic review aims to provide a comprehensive description of the most recent published research on the effects of para-aortic lymphadenectomy on overall survival, disease-free survival, and post-operative complications in patients with ovarian, endometrial, gastric, and bladder cancer.

## 2. Materials and Methods

Preferred Reporting Items for Systematic Review and Meta-Analyses (PRISMA) checklist guidelines were followed [16].

### 2.1. Search Strategy

Five different electronic databases, including PubMed, Cochrane Library, Clinical trials.gov, ICTRP, and Embase, were used to conduct a comprehensive literature search. However, there were constraints in terms of language or time of publication. Therefore, only articles in English were retrieved. Editorials, conference proceedings, and practice guidelines were not included in this study.

The searches used the PICO (P: patient or problems; I: intervention; C: comparison of interventions; O: outcome measurement) strategy. The key terms used for database searches were ovarian cancer OR cervical cancer OR endometrial cancer OR gastric cancer OR bladder cancer AND para-aortic lymphadenectomy AND overall survival OR disease-free survival, and only original research articles were retrieved and reviewed.

This study was registered under PROSPERO ID: CRD42024335349.

### 2.2. Study Selection

The titles and abstracts were reviewed for eligibility criteria after duplicates were removed. Relevant articles were selected, data were extracted, and the quality was assessed. The abstracts and full-text papers of identified studies were independently reviewed (G.V). Data were extracted in predesigned form, following which, they were synthesized.

### 2.3. Criteria for Considering Studies

The inclusion criteria included published randomized controlled trials reporting on para-aortic lymphadenectomy in ovarian, uterine, gastric, and bladder cancers. The exclusion criteria were (1) articles not related to the study; (2) studies not providing sufficient data or results; (3) studies published in languages other than English; (4) commentaries, guidelines, editorials, book chapters, letters to editors, reviews, and metanalyses; (5) animal studies; (6) other types of cancers; (7) protocols; (8) and non-randomized trials.

### 2.4. Data Extraction and Synthesis

Data were retrieved from selected research articles by an independent reviewer (G.V.). Any disagreements were sorted out through discussion. A standard Excel worksheet was used to extract the data. The overview and key characteristics of the included studies are presented in Table 1. Data on the authors, year of publication, country, sample size, type of cancers, primary endpoints, and outcomes of study were collected for each included study.

### 2.5. Study Quality Assessment

The quality assessment of the selected studies was conducted using the Revised Cochrane Risk of Bias Tool Rob 2 for randomized trials [17,18]. The assessment of studies included evaluations of (1) the randomization process, (2) any deviations from the intended interventions, (3) the missing outcome data, (4) the measurements of the outcome, and (5) the selection of the reported results. All of the studies included were evaluated for the risk of bias and then classified accordingly, that is, studies with low risk and high risk of bias and studies with some concerns.

## 3. Results

### 3.1. Identification and Description of Studies

**Figure 1:**
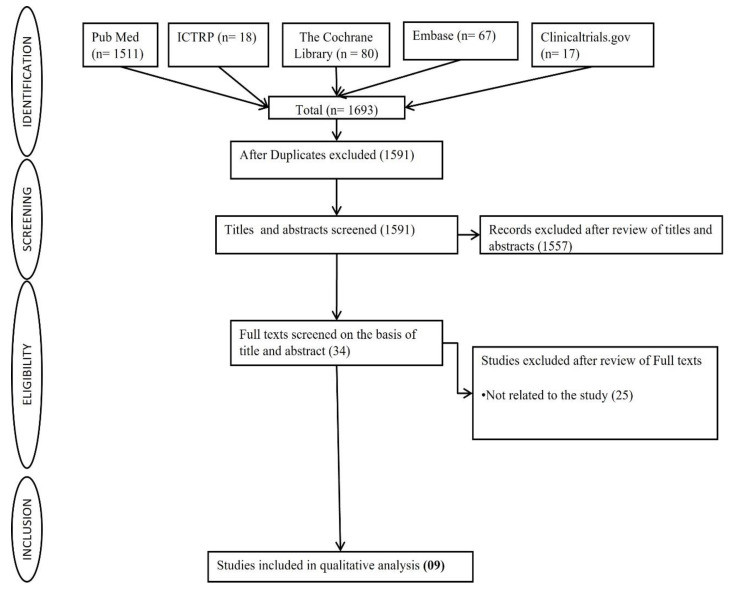
Flow chart showing the process of selecting or rejecting articles for inclusion in this study.

There were a total of 1693 citations, with 1511 from PubMed, 80 from the Cochrane Library, 67 from Embase, 18 from ICTRP, and 17 from Clinicaltrials.gov. From these, 102 duplicate studies were removed. A total of 1557 studies were eliminated after the titles and abstracts of 1591 articles were evaluated. The remaining 34 studies fulfilled the full-text review criteria. After applying exclusion criteria, 25 full texts were excluded, and the remaining 9 studies were included for final qualitative analysis. In Figure 1, the flow diagram illustrates the process of study selection.

### 3.2. Key Characteristics of the Included Studies

The key characteristics of each selected study are summarized in Table 1, and the results (overall survival, recurrence-free survival, and post-operative complications) are tabulated in Table 2. Out of the total of nine studies included in our review, three were conducted in Japan; two in Germany; and one each in Italy, Poland, the Netherlands, and the United Kingdom.

Among these nine studies, four studies were on genital cancers (two ovarian cancers, one endometrial cancer, and one cervical cancer); four on digestive cancers (advanced gastric cancers); and one on urinary cancer (advanced bladder cancer). The total number of patients was 4231, and the sample size ranged from 70 to 1408. A total of 1369 patients underwent a para-aortic lymphadenectomy versus 1641 patients who did not. In one study on endometrial cancer (ASTEC study), only pelvic lymphadenectomy was performed.

In the study by Harter et al. [3], the inclusion criteria for randomization were macroscopically negative nodes during surgery.

In the study by Panici et al. [14], the inclusion criteria were patients with epithelial ovarian carcinoma that were optimally debulked (≤1 cm) and epithelial ovarian carcinoma with FIGO Stages III and IV (only pleural effusion)

In the study by Timmers et al. [19], only patients in the observational group with EOAC were allocated to have optimal staging with para-aortic lymphadenectomy or not.

In the study by Kitchener et al. [2], patients with histologically proven endometrial carcinoma confined to the corpus underwent standard surgery (hysterectomy and BSO, peritoneal washings, and palpation of para-aortic nodes) or standard surgery plus lymphadenectomy.

### 3.3. Effectiveness of Lymphadenectomy on the Overall Survival, Progression-Free Survival, Morbidity, and Mortality in Different Cancers

The primary endpoint of the selected studies was overall survival, recurrence-free survival, disease-free survival, and the mortality rate among subjects who underwent lymphadenectomy versus those who did not.

### 3.4. Overall Survival (OS)

#### 3.4.1. Genital Cancers

According to Harter et al. [3] (advanced ovarian cancers), the overall survival was 65.5 months for those who underwent lymphadenectomy and 69.2 months for those who did not. The lymphadenectomy group had a 1.06 hazard ratio for mortality (*p* = 0.65). According to Panici et al. [14] (ovarian cancers), the groups with no lymphadenectomy and systematic lymphadenectomy had overall survival rates of 56.3 months and 58.7 months, respectively. However, after a follow-up of 68.4 months, they noted 292 events (i.e., recurrences or mortalities).

Timmers et al. [19] (early ovarian cancers) found that patients in the optimally staged group had considerably higher 5-year disease-free survival (DFS) (*p* = 0.03) rates (79% vs. 61%) and 5-year overall survival (*p* = 0.01) rates (87% vs. 71%) than patients in the other group who had undergone all staging procedures but not para-aortic or pelvic lymph node sampling. Both the disease-free survival (*p* = 0.02) and overall survival (*p* = 0.003) rates significantly differed between the patients in the optimally staged group and those in the group who underwent all staging procedures other than blind peritoneal biopsies.

After a follow-up of 37 months, Kitchener et al. [2] (endometrial cancers) reported that 191 women (88 in the regular surgery group and 103 in the lymphadenectomy group) had died, with a hazard ratio of 1.16 (*p* = 0.31) favoring standard surgery. Further, 251 women (107 who underwent conventional surgery and 144 who underwent lymphadenectomy) with an HR of 1.35 (*p* = 0.017) favored routine surgery.

#### 3.4.2. Gastric Cancers

In the study by Yonumera et al. [20] (advanced gastric cancer), two patients (0.8%, 2/256) died within 30 days of surgery, and both belonged to the D2 (Level 1 and 2 lymphadenectomies) and D4 groups (D2 plus lymphadenectomy of para-aortic lymph nodes). Kulig et al. [21] found that the total morbidity rates in the D2 (27.7%) and D2+ (plus para-aortic lymphadenectomy, 21.6%) groups were comparable (*p* = 0.248). Maeta et al. [22] found that patients in the D4 group had a postoperative hospital stay longer by 50 days than that of the D3 group (38 days). Additionally, they showed that the D4 group had a higher postoperative morbidity. Sasako et al. [15] reported overall survival rates of 69.2% and 70.3% for D2 lymphadenectomy alone and D2 lymphadenectomy with PAND, respectively.

#### 3.4.3. Bladder Cancers

Gschwend et al. [9] reported overall survival rates in patients with bladder cancer who had either limited (pelvic nodes) or extended lymph node dissection (LND). They found that neither overall survival (59% vs. 50%) nor cancer-specific survival (76% vs. 65%) was improved by extended LND over limited LND.

### 3.5. Progression-Free Survival

#### 3.5.1. Genital Cancers

According to Harter et al. [3], in both the lymphadenectomy and no-lymphadenectomy groups, the median progression-free survival was 25.5 months. In the lymphadenectomy group, the hazard ratio for progression or death was 1.11 (*p* = 0.29). According to Panici et al. [14], the progression-free survival was 22.4 months in the group receiving no lymphadenectomy and 29.4 months in the group receiving a thorough lymphadenectomy. Additionally, they found that the systematic lymphadenectomy arm had a considerably decreased probability of the first incident (*p* = 0.01).

Timmers et al. [19] found that individuals with optimal staging had a 5-year DFS rate of 79% compared to 61% and 64% in other groups with patients who had complete staging except for para-aortic or pelvic lymph node biopsy or blind peritoneal biopsies, respectively. The 5-year OS reduced from 89% in the group with the optimal staging to 71% in the group with patients who had had all staging except para-aortic or pelvic lymph node biopsy and to 65% in the group with patients who had undergone all staging but blind peritoneal biopsies.

#### 3.5.2. Gastric Cancers

Sasako et al. [15] found no significant differences between the D2 lymphadenectomy alone group and the D2 lymphadenectomy plus PAND group in terms of recurrence-free survival.

#### 3.5.3. Bladder Cancers

According to Gschwend et al. [9], there was no significant difference between extended LND and limited LND in terms of recurrence-free survival (RFS; 65% vs. 59%, respectively).

### 3.6. Postoperative Complications

#### 3.6.1. Genital Cancers

According to Harter et al. [3], the lymphadenectomy group reportedly experienced higher problems. The incidence of repeat laparotomies was found to be considerably higher (12.4% vs. 6.5%; *p* = 0.01), and the mortality rate within 60 days of surgery was 3.1% vs. 0.9% (*p* = 0.049). According to Panici et al. [13], the risk of mortality was comparable in the 58.7-month systematic lymphadenectomy arm and the no-lymphadenectomy group (HR = 0.97, *p* = 0.85). The median operating time was longer, and more patients in the systematic lymphadenectomy arm group needed blood transfusions.

Recurrences were seen in 11 (14.6%) of the 75 patients in the group with optimal staging, in 16 (34.8%) of the 46 patients in the group with all staging but para-aortic or pelvic lymph node sampling, and in 5 (35.7%) of the 14 patients in the group with all staging except blind peritoneal biopsies [19].

According to Kitchener et al. [2], the risk ratio was 1.04 (*p* = 0.83) for overall survival and 1.25 (*p* = 0.14) for recurrence-free survival.

#### 3.6.2. Gastric Cancers

According to Yonumera et al. [20], the most frequent consequences were anastomotic leakage, pancreatic fistula, and stomach abscess, which were not linked to lymphadenectomy. Further, the operation time and the blood loss were much more in D4 gastrectomy than in D2 gastrectomy. Medical complications occurred in the D2 and D4 groups on average at 4% and 2% each, but surgical complications occurred in 22% and 38% of patients following D2 and D4 gastrectomy, respectively. According to Kulig et al. [21], the postoperative death rates were 4.9% and 2.2%, respectively. In the D4 group, Maeta et al. [22] found that four patients experienced extended diarrhea, and four others suffered postoperative intra-abdominal fluid retention (lymphorrhea). Postoperative complications resulted in the mortality of one patient in each group (D4 and D3) even though the D4 group’s postoperative morbidity was also shown to be higher. Sasako et al. [14] reported a rate of 20.9% and 28.1% for surgery-related problems (*p* = 0.07) in the D2 lymphadenectomy alone group and the D2 lymphadenectomy plus PAND group, respectively.

#### 3.6.3. Bladder Cancers

According to Gschwend et al. [9], in the prolonged LND group, Grade 3 lymphoceles were more prevalent within 90 days of surgery.

**Table 1:** Characteristics of the included studies.

S. No.	Author	Year	Country	Type of Study	Sample Size	Type of Cancer	Primary Endpoint	Randomization
1	Harter et al. [3], LION, NCT00712218 (https://clinicaltrials.gov/study/NCT00712218, accessed on 28 September 2024)	2019	Germany	RCT	647	Advanced ovarian cancer	Overall survival	Lymphadenectomy in 323 vs. no lymphadenectomy in 324
2	Panici et al. [14]	2005	Italy	RCT	427	Advanced ovarian cancer	Progression-free and overall survival	216 systematic pelvic and para-aortic lymphadenectomy, and 211 resection of bulky nodes only
3	Timmers et al. [19]	2010	Netherlands	RCT	224	Early ovarian cancer	Disease-free and overall survival	Group A: 75 optimally staged Group B: 46 patients with all staging except para-aortic or pelvic lymph node sampling Group C: 14 patients with all staging except blind peritoneal biopsies
4	Kitchener et al. [2] ISRCTN 16571884.	2009	United Kingdom	RCT	1408	Endometrial cancer	Overall survival	704 subjects: hysterectomy and bilateral salpingo-oophorectomy, peritoneal washings, and palpation of para-aortic nodes 704 subjects: standard surgery plus lymphadenectomy
5	Yonemura et al. [20]	2006	Japan	RCT	256	Advanced gastric cancer	Morbidity and mortality	D2 (Level 1 and 2 lymphadenectomy) = 128 D4 (D2 plus lymphadenectomy of para-aortic lymph nodes) = 128
6	Kulig et al. [2,21]	2007	Poland	RCT	275	Advanced gastric cancer	Benefits of extended D2 lymphadenectomy	Lymphadenectomy 141 D2 (standard) vs. 134 D2+ (extended)
7	Maeta et al. [22]	1999	Japan	RCT	70	T3 or T4 gastric cancer	Overall survival	Lymphadenectomy 35 D4 (Group A) vs. 35 D3 (Group B)
8	Sasako et al. [15], NCT00149279	2008	Japan	RCT	523	Gastric cancer	Overall survival	263 patients: D2 lymphadenectomy alone 260 patients: D2 lymphadenectomy plus PAND
9	Gschwend et al. [9] NCT01215071 (https://clinicaltrials.gov/study/NCT00149279, accessed on 28 September 2024)	2019	Germany	RCT	401	Bladder cancer	Overall survival	Randomization to limited 203 (pelvic nodes) vs. 198 extended lymph node dissection

**Table 2:** Effectiveness of lymphadenectomy on the overall survival and the post-operative complications. Key findings detailed from the included studies.

S. No.	Author	Year	Median Overall Survival	Median Progression-Free Survival	Postoperative Complications	Conclusion
1	Harter et al. [3]	2019	69.2 months for those who underwent lymphadenectomy and 65.5 months for those who did not. Death hazard ratio for those who had lymphadenectomy: 1.06; *p* = 0.65.	In both groups, the median progression-free survival was 25.5 months. In the lymphadenectomy group, the hazard ratio for progression or death was 1.11 95% CI; 0.92 to 1.34; *p* = 0.29.	More complications in the lymphadenectomy group. Incidence of repeat laparotomy, 12.4% vs. 6.5% [*p* = 0.01]. Mortality within 60 days after surgery, 3.1% vs. 0.9% [*p* = 0.049]).	Pelvic and paraaortic lymphadenectomy led to a greater incidence of postoperative complications and was not linked to a longer overall or progression-free survival.
2	Panici et al. [14]	2005	56.3 months for those who underwent a systematic lymphadenectomy and 58.7 months for those who did not. After a median follow-up of 68.4 months, 292 events (i.e., recurrences or deaths) were observed, and 202 patients had died.	22.4 months for those who underwent no lymphadenectomy and 29.4 months for those who underwent systematic lymphadenectomy. The systematic lymphadenectomy arm’s risk for the first event was considerably reduced (HR = 0.75, [CI] = 0.59 to 0.94; *p* = 0.01).	In both groups, the risk of mortality was comparable (HR = 0.97; 95% CI = 0.74 to 1.29; *p* = 0.85). The operating time in systematic lymphadenectomy arm was longer, and a larger percentage of patients needed blood transfusions.	When advanced ovarian cancer has been optimally debulked, systemic lymphadenectomy increases progression-free but not overall survival.
3	Timmers et al. [19]	2010	Compared to Group B, Group A had significantly better 5-year DFS (*p* = 0.03) and 5-year OS (*p* = 0.01). Additionally, a significant difference between Group A and Group C was seen in the 5-year DFS (*p* = 0.02) and 5-year OS (*p* = 0.003).	In Group A, the 5-year DFS was 79%, compared to 61% and 64% in Groups B and C, respectively. In Groups B and C, the 5-year OS dropped from 89% in Group A to 71% and 65%, respectively.	11 (14.6%) of the 75 patients in Group A, 16 (34.8%) of the 46 patients in Group B, and 5 (35.7%) of the 14 patients in Group C experienced recurrences.	Individuals who underwent para-aortic and pelvic lymph node sampling, as well as the taking of blind peritoneal biopsies, showed statistically significant differences from the patients in whom these staging procedures had not been performed.
4	Kitchener et al. [2]	2009	191 women (88 in the standard surgery group and 103 in the lymphadenectomy group) had died after a median follow-up of 37 months, with a hazard ratio of 1.16 (95% CI 0.87–1.54; *p* = 0.31) favoring standard surgery.	251 women died or had recurrent illness (107 underwent conventional surgery and 144 underwent lymphadenectomy), with an HR of 1.35 (1.06–1.73; *p* = 0.017) favoring routine surgery.	The HR was 1.04 (0.74–1.45; *p* = 0.83) for overall survival and the HR was 1.25 for recurrence-free survival (0.93-1.66; *p* = 0.14).	Pelvic lymphadenectomy did not improve overall or recurrence-free survival.
5	Yonemura et al. [19]	2006	Two patients (0.8%, 2/256) died after 30 days of surgery, and each belonged to the D2 and D4 groups.		The D4 gastrectomy took substantially longer to perform and resulted in more blood loss than the D2 gastrectomy. In the D2 and D4 groups, medical problems occurred at a rate of 4% and 2%, respectively. Following D2 and D4 gastrectomy, surgical complications occurred in 22% and 38% of cases.	In D4 dissection, the risk of surgical complications is much greater. When carried out by skilled surgeons, D4 dissection may be done as safely as D2 dissection.
6	Kulig et al. [2,21]	2007	Overall morbidity rates in the D2 (27.7%) and D2+ (21.6%) groups were comparable (*p* = 0.248).		4.9% and 2.2% of patients died after surgery, respectively in D2 and D2+ (*p* = 0.376).	Regarding the degree of lymph node dissection, there was no substantial difference.
7	Maeta et al. [22]	2009	Patients in Group A spent 50 days in the hospital following surgery compared to 38 days for the Group B patients.	Postoperative morbidity for Group A was greater.	In group A, 4 patients experienced prolonged diarrhea, while 4 others suffered postoperative intra-abdominal fluid retention (lymphorrhea). Each group had one patient who died from complications after surgery.	The difference in postoperative survival following D4 resection between the groups was not statistically significant.
8	Sasako et al. [15]	2008	Group D2 lymphadenectomy alone had an overall survival of 69.2%, whereas the Group D2 lymphadenectomy plus PAND had a survival of 70.3%. The risk of mortality was 1.03 (*p* = 0.85) for both groups.	There were no considerable differences in the recurrence-free survival between groups.	Surgery-related problems occurred in 20.9% of patients who underwent D2 lymphadenectomy and 28.1% of patients who underwent D2 lymphadenectomy plus PAND (*p* = 0.07).	Treatment with D2 lymphadenectomy with PAND did not increase the survival rate in patients with curable gastric cancer compared to D2 lymphadenectomy alone.
9	Gschwend et al. [9]	2019	In terms of cancer-specific survival (CSS 76% vs. 65%) and overall survival (OS 59% vs. 50%), extended LND was not superior than limited LND.	Regarding recurrence-free survival, extended LND did not outperform limited LND (RFS: 65% vs. 59%).	Within 90 days of surgery, Clavien Grade 3 lymphoceles were more commonly observed in the prolonged LND group.	In RFS, CSS, and OS, the extended LND was not able to demonstrate a substantial benefit over limited LND.

### 3.7. Study Quality Assessment

**Figure 2:**
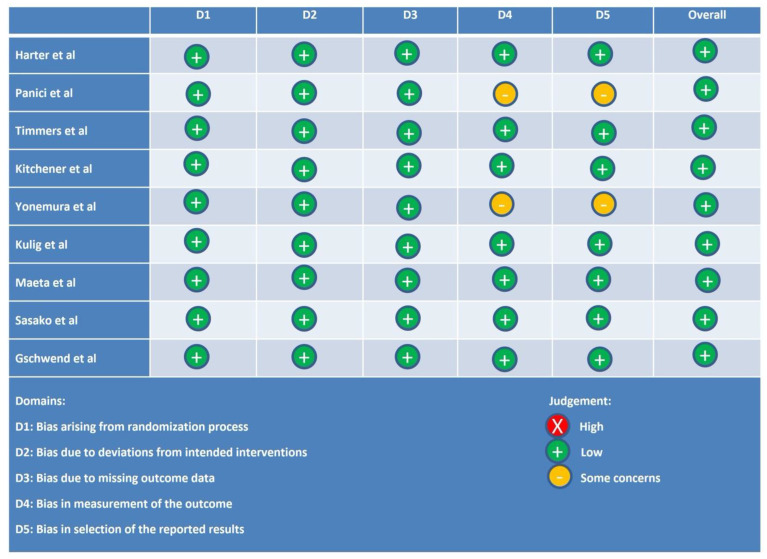
Cochrane risk of bias assessment for each component across all the studies.

The quality of the included studies was independently assessed by the two reviewers using the RoB 2 assessment tool. All of the RCTs included in this analysis had a low risk of bias. Figure 2 represents the Cochrane risk of bias assessment for each component across all the studies.

## 4. Discussion

Para-aortic lymphadenectomy is a common surgical procedure that serves both diagnostic and therapeutic purposes. It helps in staging, offers prognostic information, and affects the patient’s adjuvant therapy options. For optimal ovarian cancer debulking and staging of endometrial cancer, lymphadenectomy may be suggested. Although lymphadenectomy is not a part of cervical cancer staging, it can offer prognostic information [1,11]. However, there is still contention over the potential of para-aortic node dissection in treating cancer. The effectiveness of para-aortic lymphadenectomy on overall survival, progression-free survival, and mortality in several cancer types, such as ovarian cancer, endometrial cancer, advanced gastric cancer, and bladder cancer, was assessed in this thorough evaluation of the published literature.

Para-aortic lymphadenectomy did not increase overall survival or disease-free survival in patients with ovarian cancer in Stages IIB to IV, as shown by two RCTs. According to Harter et al. [3], systematic pelvic and para-aortic lymphadenectomy has been linked to a greater frequency of postoperative complications and is not related to a longer overall or progression-free survival than no lymphadenectomy. However, Panici et al. [14] observed that, in women with optimally debulked advanced ovarian cancer, thorough lymphadenectomy increases progression-free survival but not overall survival rate. In cases of recurrences, including lymph node metastasis after secondary cytoreduction, a tertiary cytoreductive surgery was associated with improved overall survival and disease-free survival than suboptimal surgery [23,24,25,26]. Bruno et al. [27] reported laparoscopic tertiary cytoreductive surgery for a 54-year-old patient. After 16 months, no recurrence was detected. This experience confirms that tertiary cytoreductive surgery can be considered an effective therapeutic option for ILRN management, even in patients with BACR mutations and who have already treated with PARPi. The achievement of a complete cytoreduction must be the aim of the treatment of this kind of recurrence.

However, Timmers et al. [19] discovered that individuals who underwent para-aortic and pelvic lymph node sampling, as well as the collection of blind peritoneal biopsies, had statistically significant differences from patients in whom these staging stages had been omitted. Lymphadenectomy may improve overall survival and disease-free survival rates in early-stage epithelial ovarian cancers when adjuvant chemotherapy is not indicated [27,28].

In some cases, complete para-aortic lymphadenectomy could be replaced by sentinel lymph node dissection, which is a less invasive technique. Sentinel lymph node dissection in early-stage ovarian cancers has a high detection rate [29]. In the case of early endometrial cancers localized to the uterus, there are no benefits in terms of overall survival or DFS, even if the histological type is considered. According to Kitchener et al. [2], in women with early endometrial cancer, pelvic lymphadenectomy did not appear to improve overall or recurrence-free survival. Therefore, pelvic lymphadenectomy cannot be advised as a standard treatment technique outside of controlled studies. Studies have also shown that para-aortic lymphadenectomy did not improve disease-free survival or overall survival in surgical patients with Stage IB1-IIA2 cervical cancer [30]. In early endometrial cancers with high risk, sentinel lymph node dissection became an alternative to complete lymphadenectomy [31]. Indeed, the sensitivity of detection of the sentinel lymph node is high when using a different colloid tracer (e.g., blue dyes or indocyanine green) labeled with radioactive technetium-99 [31].

For advanced gastric cancers, D4 lymphadenectomy did not show any benefit over D2 alone concerning OS and DFS, and the complications were significant, especially regarding when more transfusions were conducted due to vascular injuries and lymphedema. Therefore, a systematic D4 lymphadenectomy should not be performed. According to Maeta et al. [22], there was no statistically significant difference between the groups in terms of post-operative survival following D4 resection. Yonemura et al. [20] observed a considerably greater prevalence of surgical complications in D4 dissection. Therefore, it was advised that D4 dissection be carried out by qualified surgeons in the same way as D2 dissection. In terms of the degree of lymph node dissection, Kulig et al. [21] discovered no significant difference. Sasako et al. [15] found that, compared to D2 lymphadenectomy alone, therapy with PAND failed to increase the survival rate in patients with curable gastric cancer.

The risk of radiation-related morbidity can be decreased by lymphadenectomy by identifying individuals who have metastatic spread and who could benefit from adjuvant treatment, thus reducing the rate of unnecessary treatment and associated morbidity [32]. However, a study revealed that preoperative chemotherapy followed by PAND is recommended in individuals with clinically diagnosed para-aortic nodal metastases [33]. In some instances, despite the rising amount of data supporting LND in radical cystectomy, the direct therapeutic impact is still difficult to discern from the available literature, making it difficult to make definitive recommendations [34]. Although the extent of lymph node dissection is still debatable, an increasing number of studies is demonstrating the additional diagnostic and therapeutic benefits of an extended lymphadenectomy [10].

All of the studies in this comprehensive review showed that systematic para-aortic lymphadenectomy is not advised in genital malignancies, stomach cancers, or bladder tumors, depending on their various phases. It is contraindicated due to several major post-operative complications and a lack of solid evidence of its benefits. Additionally, a comprehensive para-aortic lymphadenectomy removes not just potentially cancerous nodes, but also healthy nodes that serve as an immune defense against cancer cells. Therefore, patients should be educated adequately on the procedure before subjecting them to such invasive operations because the effectiveness of lymphadenectomy is not proven, and the risks are substantial. New RCTs are required to demonstrate any benefits on the overall survival in cancers at other stages.

Limitations of this study: The number of studies reviewed was small. However, all of the studies included in this analysis were RCTs and had a low risk of bias. In the majority of the studies, para-aortic lymphadenectomy was performed by laparotomy because it was a part of the surgical procedure. Therefore, a comparison between the complications of para-aortic lymphadenectomy in the case of open and laparoscopic or robotic surgery was not reported in the examined studies.

## 5. Conclusions

Keeping in view the frequent intraoperative and postoperative problems—including extended operative time, increased blood loss, the need for transfusion because of vascular injuries, lymphedema of the lower limbs, higher rates of morbidity and mortality, and no advantage in terms of overall survival—the indications for complete para-aortic lymphadenectomy become rare.

In advanced ovarian malignancies, early low-risk endometrial cancers, advanced gastric cancers, and advanced bladder cancers, para-aortic lymphadenectomy did not increase overall survival or disease-free survival rate and is, therefore, not recommended. For early ovarian cancers and high-risk endometrial cancers, sentinel lymph node biopsy could be an alternative to lymphadenectomy.

Even in these cases, informed consent of the patients should be obtained only after explaining not only the potential survival benefits, but also the potential complications and risks of this procedure.
